# Characterization and Comparison of the Structural Features, Immune-Modulatory and Anti-Avian Influenza Virus Activities Conferred by Three Algal Sulfated Polysaccharides

**DOI:** 10.3390/md14010004

**Published:** 2015-12-29

**Authors:** Lin Song, Xiaolin Chen, Xiaodong Liu, Fubo Zhang, Linfeng Hu, Yang Yue, Kecheng Li, Pengcheng Li

**Affiliations:** 1 Key Laboratory of Experimental Marine Biology, Institute of Oceanology, Chinese Academy of Sciences, No.7 Nanhai Road, Qingdao 266071, China; lylinsong@hotmail.com (L.S.), wood9818@sina.com (L.H.), yueyang12@ucas.ac.cn (Y.Y.); lkc@qdio.ac.cn (K.L.); 2 University of Chinese Academy of Sciences, Beijing 100049, China; 3 College of Animal Science and Technology, Qingdao Agriculture University, No.700 Changcheng Road, Qingdao 266109, China; lxdau86@163.com (X.L.); tsygy@whu.edu.cn (F.Z.)

**Keywords:** marine macroalgae, sulfated polysaccharides, structure features, immune enhancement, anti-AIV

## Abstract

Three marine macroalgae, *i.e.*, *Grateloupia filicina*, *Ulva pertusa* and *Sargassum qingdaoense*, were selected as the deputies of Rhodophyta, Chlorophyta and Ochrophyta for comparative analysis of the molecular structures and biological activities of sulfated polysaccharides (SP). The ratio of water-soluble polysaccharides, the monosaccharide composition and the sulfated contents of three extracted SPs were determined, and their structures were characterized by Fourier transformation infrared spectroscopy. In addition, biological activity analysis showed that all three SPs had immune-modulatory activity both *in vitro* and *in vivo*, and SPs from *S. qingdaoense* had the best effect. Further bioassays showed that three SPs could not only enhance the immunity level stimulated by inactivated avian influenza virus (AIV) *in vivo* but also significantly inhibited the activity of activated AIV (H9N2 subtype) *in vitro*. *G. filicina* SP exhibited the strongest anti-AIV activity. These results revealed the variations in structural features and bioactivities among three SPs and indicated the potential adjuvants for immune-enhancement and anti-AIV.

## 1. Introduction

Sulfated polysaccharides have attracted increasing attention due to their diversified biological and pharmacological activities, such as anti-viral, immune enhancement, anti-tumor, anti-infective and antioxidant effects (Table 1) [1,2,3,4]. Among organisms that produce sulfated polysaccharides, marine algae are regarded as the most abundant and important sources of non-animal sulfated polysaccharides [5,6,7]. Various biological functions of sulfated polysaccharides from marine algae have been reported in recent decades, but these analyses were mainly focused on a single species (Table 1) [8,9,10,11]. For example, the sulfated polysaccharides obtained from green alga *Enteromorpha clathrata* showed immune-enhancement activity that could stimulate TNF-α expression in serum and induce lymphocyte proliferation [9]. The sulfated polysaccharide purified from *Sargassum horneri* was reported to have antitumor activity that could inhibit the growth of human colon cancer DLD cells [12]. The *S. vulgare* polysaccharides illustrated an ability to enhance serum antibody titers and lymphocyte proliferation [10]. Furthermore, the sulfated polysaccharides extracted from the red alga *Laurencia papillosa* could inhibit breast cancer cells (MDA-MB-231) via apoptosis regulatory genes [13]. While significant attention has been paid to one or two biological and pharmacological activities of sulfated polysaccharides from a single species of marine algae, little information is available regarding the bioactivity comparison of sulfated polysaccharides from different algae and the structure-function relationship. The three main divisions of marine algae (*i.e.*, Chlorophyta, Ochrophyta and Rhodophyta) are valuable sources of structurally diverse sulfated polysaccharides. However different sulfated polysaccharides from these three algae still remain largely unknown in the comparative analysis of the molecule structures and diverse bioactivities.

**Table 1:** Previous studies on the biological effects of sulphated polysaccharides from seaweed.

Author	Phylum	Species	Bioactivities
Qi, *et al.*, 2005 [14]	Chlorophyta	*Ulva pertusa*	Antioxidant activity
Zhang *et al.*, 2010 [2]		*Ulva pertusa*	Antioxidant activity
		*Enteromorpha linza*	
		*Bryopsis plumose*	
Cho *et al.*, 2010 [15]		*Enteromorpha prolifera*	Antitumor and immunomodulating activities
Jiao *et al.*, 2010 [16]		*Enteromorpha intestinalis*	Antitumor and immunomodulating activities
Tabarsa *et al.*, 2012 [17]		*Ulva pertusa*	Immunomodulatory, anticancer activities
Zhang *et al.*, 2013 [18]		*Enteromorpha linza*	Immunological and antioxidant activities
Aguilar-Briseño *et al.*, 2015 [19]		*Ulva clathrata*	Antiviral activity
Zhang *et al.*, 2010 [2]	Ochrophyta	*Laminaria japonica*	Antioxidant activity
Ye *et al.*, 2008 [20]		*Sargassum pallidum*	Antitumor and antioxidant activities
Wang *et al.*, 2011 [21]		*Laminaria japonica*	Anticoagulant activity
Li *et al.*, 2012 [22]		*Sargassum pallidum*	Immune responses
Dore *et al.*, 2013 [10]		*Sargassum vulgare*	Anticoagulant, antithrombotic, antioxidant and anti-inflammatory effects
Suresh *et al.*, 2013 [23]		*Sargassum plagiophyllum*	Anticancer and antioxidant activities
Imbs *et al.*, 2014 [24]		*Fucus evanescens*	Antioxidant activity
Hwang *et al.*, 2015 [25]		*Sargassum hemiphyllum*	Anti-inflammatory
Wen *et al.*, 2014 [26]		*Sargassum horneri*	Antioxidant activity
Shao *et al.*, 2014 [27]		*Sargassum horneri*	Antioxidant and antitumor activities
Shobharani *et al.*, 2014 [28]		*Sargassum* sp.	Antioxidant and anticoagulant activities
Aguilar-Briseño *et al.*, 2015 [19]		*Cladosiphon okamuranus*	Antiviral activity
Zhang *et al.*, 2014 [29]		*Ascophyllum nodosum*	Induces Th1 and Tc1 Immune Responses
Yuan *et al.*, 2015 [30]		*Ascophyllum nodosum*	Antioxidant activity
Ammar *et al.*, 2015 [31]		*Cystoseira sedoides,*	Anti-radical, anti-inflammatory and gastroprotective activities
		*Cystoseira compressa,*	
		*Cystoseira crinita*	
Shao *et al.*, 2015 [32]		*Sargassum horneri*	Antioxidant and moisture-preserving activities
Athukorala *et al.*, 2005 [33]	Rhodophyta	*Grateloupia filicina*	Antioxidant activity, protecting ability for H_2_O_2_-induced DNA damage
Wang *et al.*, 2007 [34]		*Grateloupia longifolia*	Anti-virus activity
		*Grateloupia filicina*	
Zhang *et al.*, 2010 [2]		*Porphyra haitanensis*	Antioxidant activity
Yu *et al.*, 2012 [35]		*Eucheuma denticulatum*	Anti-virus activity
Shi *et al.*, 2014 [36]		*Porphyra haitanensis*	Anti-allergic activity
Chen *et al.*, 2015 [37]		*Grateloupia filicina*	Anticoagulant activity
Fleita *et al.*, 2015 [38]		*Pterocladia capillacea*	Antioxidant activity

Sulfated polysaccharides from algae produce immune-modulatory activities that might have great potential for stimulating immune responses or controlling immune cell activity [8]. Karnjanapratum reported that the biological activities of water-soluble sulfated polysaccharides isolated from *Monostroma nitidum* could stimulate Raw 264.7 cells *in vitro*, and induce considerable prostaglandin-2 (PGE-2) and nitric oxide (NO) production [39]. Oral ingestion of polysaccharides isolated from *E. intestinalis* could increase the relative spleen and thymus weight of tumor-bearing animals and stimulate lymphocyte proliferation *in vitro* [16]. In addition to immune-modulatory activity, the antiviral activity of sulfated polysaccharides is also important [40]. It has been suggested that sulfated polysaccharides from algae confer activities that are anti-viral to herps simplex virus type 1 (HSV-1), herps simplex virus type 2 (HSV-2), and human immunodeficiency virus (HIV) [5].

As a lowly pathogenic avian influenza virus (AIV) group, H9N2 subtype influenza virus is considered to be the common cause of disease epidemics [41,42]. Additionally, outbreaks of H9N2 are associated with significant economic loss in the chicken industry [43,44]. More seriously, this subtype is characterized by cross-species infections and has been passed to pigs, ferrets and guinea pigs as well as to humans, in a small number of cases [45,46,47,48,49]. These cross-species infections indicate a potentially serious threat to human health [50,51]. The first human infection was detected in 1999, and ever since there have been several reports about the isolation of H9N2 viruses from humans and swine [52] including the latest H9N2 human case in China at the end of 2013 [53]. Sulfated polysaccharides were considered to be novel sources of natural compounds for antiviral drug discovery, but whether they could confer antiviral activity to H9N2 AIV remains elusive [54,55,56].

In this study, three marine algae, *i.e.*, *Grateloupia filicina*, *Ulva pertusa* and *Sargassum qingdaoense*, were selected as the deputies of Rhodophyta, Chlorophyta, and Ochrophyta for comparative analysis of the molecular structures and immune-modulatory and anti-AIV activities of sulfated polysaccharides. The variations in chemical compositions and molecular structures of three sulfated polysaccharides, including polysaccharide components and sulfate contents, might function as determinants of their bioactivities [17,34,57]. Thus, it is worth making an effort to analyze and determine the structure-function relationship of these sulfated polysaccharides. Here, we report a comprehensive analysis of structural features and immune-modulatory and anti-AIV activities of sulfated polysaccharides from three types of marine algae, *i.e.*, *S. qingdaoense*, *G. filicina*, and *U. pertusa.* These polysaccharides were characterized structurally and their biological activities were tested both *in vitro* and *in vivo*, which has not only enhanced our understanding of the characteristic of algae sulfated polysaccharides but also provided a comparison of algae from different categories and contributed further theoretical and experimental evidence for the exploration and development of polysaccharide-based immune-potentiators that are anti-AIV.

## 2. Results

### 2.1. Chemical Characterization of Three Sulfated Polysaccharides

#### 2.1.1. Chemical Analysis

Three sulfated polysaccharides were extracted and purified from *U. pertusa*, *G. filicina*, *S. qingdaoense*, with a yield of 12.1%, 19.7% and 7.2%, respectively (Table 2). The purified sulfated polysaccharides were further characterized regarding monosaccharide composition molar ratios. Individual variations were found in the components of monosaccharides, and the most abundant components in UPP, GFP and SQP were rhamnose, galactose, and fucose, respectively. The UPP also had glucuronic acid and xylose, and a small amount of mannose, glucose, galactose and fucose was found. For GFP, except for the main component, the content of other monosaccharides, such as mannose, glucuronic acid, glucose, xylose and fucose were low. As for SQP, which mainly consists of fucose, galactose and mannose, it also contained glucuronic acid and glucose. The ratio of total saccharides to UPP (53.13%) were also shown to be much higher than the others. Regarding sulfate contents, GFP (19.89%) was higher than UPP (13.54%) and SQP (5.64%). These results suggested significant variations in chemical compositions among the three sulfated polysaccharides.

**Table 2:** Yield and chemical composition of three sulfated polysaccharides sample (%*w/w* of dry weight).

Sample	Yield (%)	Total Sugar (%)	Sulfate (%)	Monosaccharides Composition (Molar Ratio)						
				Man	Rha	Glc A	Glc	Gal	Xyl	Fuc
UPP	12.1	53.13	13.54	0.06	1	0.53	0.19	0.09	0.39	0.02
GFP	19.7	40.9	19.89	0.01	-	0.02	0.07	1	0.1	0.05
SQP	7.2	20.81	5.64	0.56	-	0.13	0.37	0.6	-	1

Man: mannose; Rha: rhamnose; Gal: galactose; Glc: glucose; Xyl: xylose; Fuc: fucose.

#### 2.1.2. FT-IR Spectrometric Characterization

**Figure 1:**
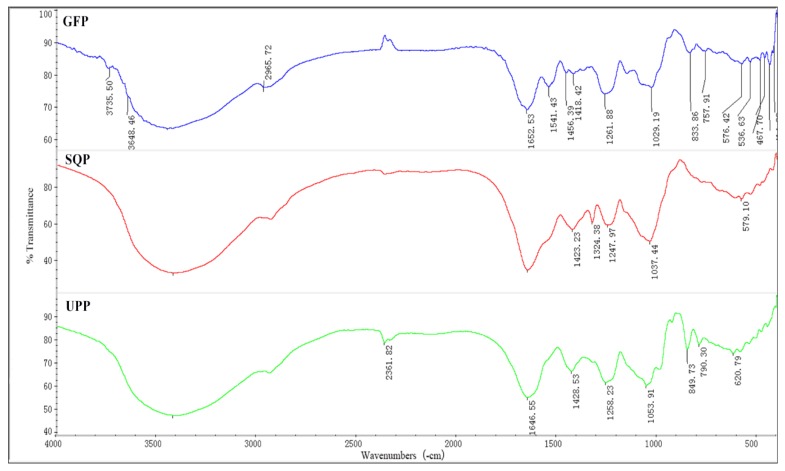
FT-IR spectra of three extracted sulfated polysaccharides. GFP, *Grateloupia filicina*; UPP, *Ulva Pertusa*; SQP, *Sargassum qingdaoens*.

To further understand the structure of sulfated polysaccharides, FT-IR spectroscopy was used. The results revealed that all three sulfated polysaccharides shared several common absorption peaks (Figure 1), which were considered to correspond to the O-H stretching vibrations (3420 cm^−1^), the S꞊O asymmetry stretching vibrations (1250 cm^−1^) the C-O-H deformation vibrations (1050 cm^−1^), and the stretching vibrations of -COO^−^ (1650 cm^−1^ and 1420 cm^−1^) which also indicated that the extraction was composed of acidic polysaccharides [58].

Additionally, there exist some differences in the FT-IR spectra of the three polysaccharides. For example, the absorption peaks at 2950 cm^−1^ for C-H stretching vibrations and at approximately 830 cm^−1^ that appeared in the FT-IR spectrum of GFP indicated the presence of α-type glycosidic linkages. The band at 1030 cm^−1^ of SQP was found and corresponded to C-O-H deformation vibrations. As for UPP, the peak attributed to C-O-S symmetry stretching vibrations appeared at approximately 850 cm^−1^ [10,34,59].

### 2.2. Cytotoxic Activity of the Polysaccharides

MTT assays to determine the cytotoxicity of sulfated polysaccharides showed that the safe concentration for GFP was 2.5 mg/mL, whereas it was 5 mg/mL for both UPP and SQP (Table A1). The concentrations of the three polysaccharides used in our experiments were all within the safe range.

### 2.3. Immunologic Modulation of Three Sulfated Polysaccharides in Vitro

**Figure 2:**
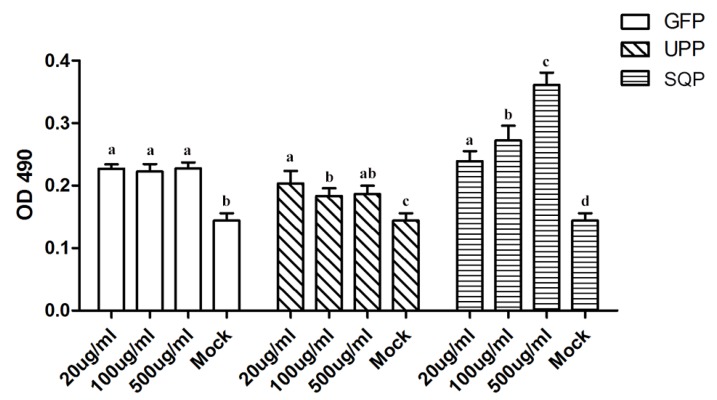
Mouse spleen cell proliferation effects of GFP, UPP and SQP. Mock treated with PBS instead of polysaccharides as a negative control. Values with different letters in the same column (a–d) are significantly different (*p* < 0.05) from each other. Data are shown as the Mean + SD and are fully representative of the individual experiment.

To understand their potential effects in immunology, these compounds were first used to test the immune response at a lymphocyte level. A lymphocyte proliferation experiment was used to evaluate the stimulation efficiency of the sulfated polysaccharides on spleen cell proliferation. As illustrated in Figure 2, the lymphocyte proliferation values for each sulfated polysaccharide treatment group were significantly higher than those of the control group (Mock; *p* < 0.05), suggesting a dramatic effect on the stimulation of spleen cell proliferation. Further analysis showed that, both UPP and GFP showed a similar effect on proliferation in response to all three treatment doses, in contrast to the fact that the stimulation efficiency of SQP was in dose-dependent manner. As a result, 500 μg/mL of SQP conferred the strongest efficiency in stimulation among all the tests.

### 2.4. Immune-Modulation of Three Sulfated Polysaccharides in Vivo

To further verify the immune-modulation results obtained by *in vitro* analysis, *in vivo* experiments were carried out in mice. Antibody titer, cytokine production and T-cell subpopulation were tested. Additionally, H9N2-AIV was selected as the immunologic stimulant.

#### 2.4.1. H9N2-Specific Antibody Titer

**Figure 3:**
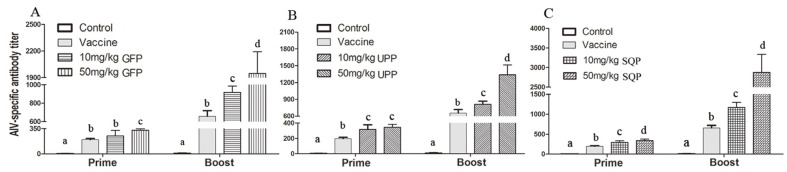
Avian influenza virus (AIV)-specific antibody titer detection. Kunming mice were immunized with an AIV vaccine and polysaccharides, following the prime-boost vaccination programme (days 0 and 14), respectively. (**A**) GFP; (**B**) UPP; (**C**) SQP. Values with different letters in the same column (a–d) are significantly different (*p* < 0.05) from each other. Data are shown as the Mean + SD and are fully representative of an individual experiment.

After the first injection (Prime), the antibody titer of the control group was almost undetectable, while the antibody levels of the treated group rose dramatically (*p* < 0.05; Figure 3). Moreover, after the second injection (Boost), the antibody titer of the control group still stayed at the base level, while further significant enhancement was detected relative to the prime for all the treatment doses especially for the case of 50 mg/kg of GFP and 50 mg/kg of SQP (Figure 3A,C). These results suggested that the assayed sulfated polysaccharides significantly increased H9N2-specific antibody titers.

#### 2.4.2. Effect on Cytokine Production Stimulation

**Figure 4:**
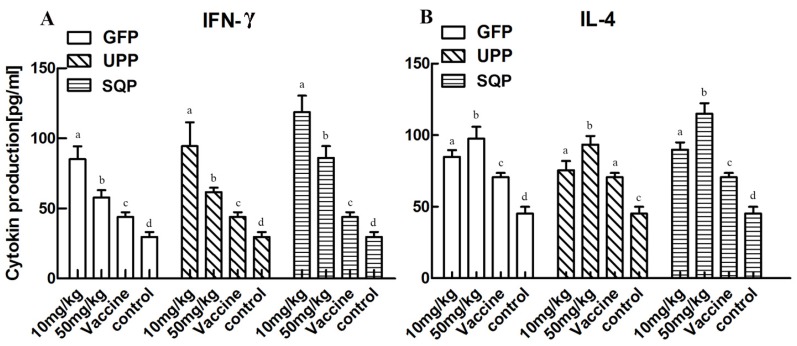
Cytokine production stimulating effect of GFP, UPP and SQP. Kunming mice were immunized with an AIV vaccine and polysaccharides, and sera were collected on day 28 after two immunizations to detect the cytokines IFN-γ (**A**) and IL-4 (**B**). Values with different letters in the same column (a–d) are significantly different (*p* < 0.05) from each other. Data are shown as the Mean + SD and are fully representative for the individual experiment.

The results of cytokine production stimulation are presented in Figure 4. The levels of IFN-γ and IL-4 were significantly increased in the experimental groups compared to the control and the vaccine groups (*p* < 0.05). However, individual variations were observed among the groups treated by three sulfated polysaccharides, and the most efficient stimulations resulted from the optimal doses tested. For example, a concentration of 10 mg/kg were more efficient for stimulation of IFN-γ production compared to that of 50 mg/kg for all three sulfated polysaccharides. By contrast, 50 mg/kg was a better concentration for the IL-4 stimulation than 10 mg/kg.

#### 2.4.3. T-Cell Subpopulation

**Figure 5:**
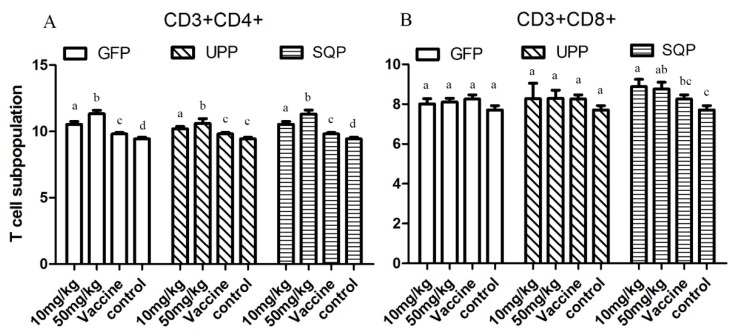
T-cell subpopulation tests. The blood cells of the treated mice were collected and analyzed with flow cytometry. (**A**) CD3+CD4+. (**B**) CD3+CD8+. Values with different letters in the same column (a–d) are significantly different (*p* < 0.05) from each other. Data are shown as the Mean + SD and are fully representative of the individual experiment.

Results presented in Figure 5 show how each of the three sulfated polysaccharides could elevate the CD3+CD4+ levels in the experimental groups significantly compared to the control and vaccine groups (*p* < 0.05), and the elevations were in a dose-dependent manner in such a way that 50 mg/kg gained a much stronger effect relative to 10 mg/kg. For stimulation of the CD3+CD8+, only the SQP stimulated groups showed a significant effect.

### 2.5. Anti-H9N2 Effect of Three Sulfated Polysaccharides in Vitro

Because all these sulfated polysaccharides could significantly enhance immune responses with the deactivated AIV as an immunologic stimulant, we were especially interested in whether they could show significant resistance to activated H9N2 AIV.

**Figure 6:**
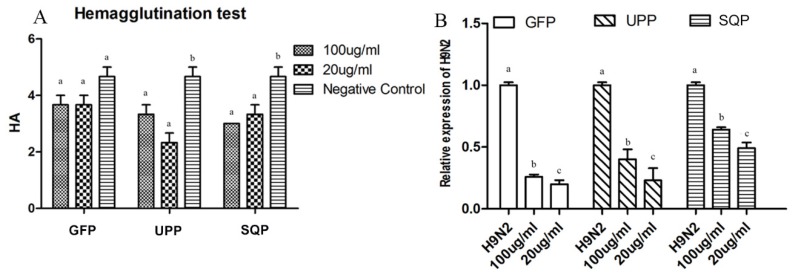
Haemagglutination test (HA) of the cell culture and relative expression of H9N2. Antiviral activity *in vitro* was measured with a HA test (**A**) and RT-PCR (**B**). Data from samples without polysaccharides were used as a basic control. Values with different letters in the same column (a–c) are significantly different (*p* < 0.05) from each other. Data are shown as the Mean + SD and are fully representative of the individual experiment.

Based on the Hemagglutination test (HA test), treatment with 0.2 mg/mL UPP and 1 mg/mL SQP in the experimental groups decreased the virus titer significantly, although the effects of other groups were not significant (Figure 6A).

From the real-time PCR results, the expression of the H9N2 gene decreased significantly after treatment with sulfate polysaccharides. GFP was the strongest of all three test groups in terms of the inhibition of H9N2 replication, and was followed by UPP. SQP showed the weakest effect compared to the other two; however, it still had a significant effect. As for the determination of the optimal concentrations for suppressing the virus, the 20 ug/mL groups had relatively lower virus expression and that was the best suppression effect compared to the other doses tested (Figure 6B).

## 3. Discussion

Sulfated polysaccharides from algae have been acknowledged to bear diversified biological and pharmacological functions [22,60,61,62]. Moreover, the chemical composition and structural features of polysaccharides were confirmed to function as determinants of their bioactivities [17,57]. Therefore, it is necessary to determine which group(s) in the molecule play(s) an essential roles in conferring the biological function(s), and this was one of our key focuses for the present study. As expected, significant variations in the chemical composition of monosaccharides, the ratio of total saccharides and sulfated content were detected among all three sulfate polysaccharides. The results were similar to the results of Zhang *et al.*, Suresh *et al.*, Dore *et al.*, Fleita *et al.* and Tabarsa *et al.* [10,14,17,23,38] The amount of saccharide is usually described using total saccharides, but there were some metal ions attached to the polysaccharide molecules (such as Ca^2+^, Na^+,^ K^+^ and Mg^2+^), and also the SO_4_^2+^; therefore, the level of total saccharides was much lower than the real saccharide amount. Moreover, the results of FT-IR showed the variations in the structural features. For example, GFP contained C-H stretching vibrations, α-type glycosidic linkages, and the highest sulfate content compare to the other two polysaccharides. Further analyses were performed to determine the structure-function relationship.

Immune-modulatory effects are some of the most important activities of polysaccharides, and they have attracted much attention and are already a focus of research [8,63]. To verify the immune-modulatory function of sulfated polysaccharides from algae, splenic lymphocyte proliferation was tested *in vitro.* It is well known that lymphocytes play important roles in the immune response; hence, the proliferation of lymphocytes becomes the most pertinent criterion to evaluate the cellular immune functions [64,65]. Our results revealed that, all three polysaccharides tested could significantly enhance spleen cell proliferation. However, it is difficult to wholly evaluate immune responses only *in vitro*; *in vivo* tests are necessary for further verification of the effect. Thus we chose mice as the model animal and selected the AIV, which is a cross-species infection, as the immunologic stimulant to examine the immune-enhancing activity of polysaccharides *in vivo*. The antibody titer is a measure of the specific humoral immune response in animals after vaccination [66]. Cytokines are believed to function as the important mediators of immune responses, such as IFN-γ, a pleiotropic cytokine with immune-modulatory effects on different types of immune cells, while IL-4 is a cytokine essential for immune-modulation [44]. IFN-γ and IL-4 were tested in the absence or presence of the polysaccharides. CD+ T lymphocytes are primarily responsible for mediating cytotoxic effects, and CD3+, CD8+ and CD4+ are important T lymphocyte markers [65]. Collectively, the results showed that the administration of UPP, GFP and SQP could significantly enhance AIV-specific antibody production and improve the humoral immunity level, and the optimal doses that most efficiently stimulated the production of immunity level varied depended on the type of algae. It was revealed that, 50 mg/kg SQP exhibited the highest AIV-specific antibody titer and IL-4, while 10 mg/kg SQP was the best activator of IFN-γ. As for the T-cell subpopulation, 50 mg/kg of GFP and SQP elicited the best efficiency for CD3+CD4+, while for CD3+CD8+, 10 mg/kg of SQP produced higher levels compared to others. In sum, SQP might have the best immune-enhancing effects among these three algae polysaccharides, particularly at 50 mg/kg. The reason why SQP showed the best humoral immune-enhancing response might be related to the most abundant monosaccharide that SQP contained, which was fucose. This result is supported by studies that showed fucose plays an important role in immune-enhancing activity [67]. Thelen *et al.* reported that fucoidin from *F. vesiculosus* had been shown to induce macrophage activation [68]. Moreover, fucose from *F. vesiculosus* could function as an effective adjuvant as reported by Jin *et al.* [69]. Our results confirmed and inferred that the fucose group in polysaccharide molecules might contribute more to the enhancement of spleen cell proliferation and humoral immune responses than other components. These results also suggested that the polysaccharides could function as enhancers in the immune responses and SQP was recommended.

As described above, because all three sulfated polysaccharides could significantly enhance the immune response with the deactivated AIV as an immunologic stimulant, we were especially interested in whether they could resist the activated AIV. In this study, the significant suppressions of virus replication and virus gene expression were detected by both the HA test and the real-time PCR, suggesting that these sulfate polysaccharides could suppress the replication and expression of AIV *in vitro*. GFP suppressed the AIV-replication and expression more significantly compared to the other two polysaccharides, which might be related to the high sulfate content of GFP. The sulfated content was thought to be related to higher anti-virus activity [54,70]. Chen *et al.* found that the antiviral activity of sulfated polysaccharides from bush sophora roots was stronger than regular polysaccharides that were not sulfated [71]. The anti-duck hepatitis A virus (DHAV) activities of sulfated polysaccharides from *Astragalus* were stronger than those of *Astragalus* polysaccharides, both *in vitro* and *in vivo* [72]. AIV is a highly contagious disease in domestic poultry and other animals, even humans. Although a vaccination is one of the most promising measures to control AIV, the high frequency of virus antigenic variation has led to difficulties in the use of the H9N2-specific vaccines [44,64,73]. Thus, it is important to seek effective and broad-spectrum antiviral drugs for the treatment of H9N2. Based on these results, these three sulfated polysaccharides are suggested to be a potential alternative to vaccine-based prevention to reduce the breakage of AIV H9N2, among which GFP is recommended.

## 4. Materials and Methods

### 4.1. Algal Samples

Both *G. filicina*and and *U. Pertusa* were collected from the No. 2 Bathing Beach of Qingdao, China. *S. qingdaoens* was preserved at IOCAS (Institute of Oceanology, Chinese Academy of Sciences, Qingdao, China). The algae were washed with distilled water, dried at 50 °C and stored at room temperature for later use (within one year). All reagents were analytical grade and commercially available.

### 4.2. Extraction of Water-Soluble Sulfated Algal Polysaccharide

Water-soluble sulfated algal polysaccharides were extracted according to the protocol reported by Zhang. *et al.* protocol with improvements [18]. The water volume, temperature and extraction time for each type of seaweed were decided according to the optimized methods before. For *G. filicina*, a 50-fold volume of water was used at 100 °C for 2 h with stirring, and for *U. Pertusa*, 4000 mL H_2_O/100 g was mixed at 125 °C for 4 h, while 100 g of *S. qingdaoens* was soaked with 30-fold water at 91 °C for 4 h with stirring. Then, the polysaccharides solution gained by filtration was condensed and dialyzed for salt removal. The solution was condensed again and freeze-dried to obtain the purified sulfated polysaccharides named UPP, GFP and SQP for *U. Pertusa*, *G. filicina,* and *S. qingdaoens*, respectively.

### 4.3. Chemical Characterization

Total carbohydrate content was analyzed with phenol–sulfuric acid method using galactose, rhamnose, and fucose as the standard for GFP, UPP, and SQP, respectively [74].

The molar ratios of monosaccharide composition were measured in reference to Zhang *et al.* [66]. Briefly, polysaccharides (10 mg/mL) were hydrolysed in trifluoroacetic acid, followed by neutralization with sodium hydroxide. Then, pre-column derivatization with 3-methyl-1-phenyl-2-pyrazolin-5-one (PMP; 99%) to neutralize the mixture was carried out and separated by HPLC on a YMC Pack ODS AQ column (4.6 mm × 250 mm) [75]. The standards for monosaccharide composition analysis and PMP were obtained from Sigma Aldrich (St Louis, MO, USA).The sulfated content was measured by the barium chloride gelatin method following Kawai *et al.* [76].

FT-IR spectra of the three types of polysaccharides were determined on a Nicolet-360 FT-IR spectrometer (36 scans, at a resolution of 6 cm^−1^) between 400 cm^−1^ and 4000 cm^−1^. The dried polysaccharide samples were grinded with potassium bromide (KBr) and pressed into pellets for spectrometric measurement [71].

### 4.4. Animals and Maintenance

Kunming mice at the age of six weeks were purchased from Qingdao Laboratory Animal Center (Qingdao, China) for this study. All animals were housed under standard environmental conditions (22 ± 0.5 °C, 55% ± 5% humidity and a 12 h light/12 h dark cycle) and maintained with free access to a standard laboratory pellet diet and water. All procedures involving animals throughout the experiments were conducted in strict accordance with the Chinese Legislation on the Use and Care of Laboratory Animals. All animal experiments were performed as per the local institutional ethic committee guidelines.

### 4.5. Cell Lines, Virus, and Tissue Culture

Madin-Darby canine kidney (MDCK) cells were purchased from American type culture collection (ATCC, Manassas, VA, USA). The cells were grown in Dulbecco’s modified Eagle’s medium (DMEM) (Corning INC., Corning, NY, USA) supplemented with 100 units/mL penicillin, 100 mg/mL streptomycin (HyClone Laboratories, Logan, Utah, USA), and 10% (*v*/*v*) fetal bovine serum (FBS; GIBCO BRL Life Technologies, Grand Island, NY, USA) for normal growth and 1% (*v*/*v*) for viral infection.

AIV H9N2 was kept in Qingdao Boite Biopharmaceutical CO., LTD Company (Qingdao, China). It was propagated on ten-day-old embryonating specific-pathogen-free (SPF) chicken eggs. Titers of the AIV H9N2 were quantified using MDCK cell monolayers by determining the 50% Tissue Culture Infective Dose (TCID50), and the 100 TCID50 of purified virus were used in the subsequent experiments.

### 4.6. Cytotoxic Activity Evaluation

An MTT assay was applied to determine the relative survival rate of cells during culture [77,78,79]. After 24 h incubation of 2.5 × 10^4^ cells/mL MDCK cells on 96-well plates either in DMEM as a control or in sulfated polysaccharides dissolved in DMEM (the concentrations was 10 mg/mL and diluted in 2-fold steps), the cells were treated with the MTT (5 mg/mL, 30 μL/well, Beijing Solarbio Science & Technology Co. Ltd., Beijing, China) reagent. After 4 h, the supernatant was removed and 100 μL of DMSO (Beijing Solarbio Science & Technology Co. Ltd., Beijing, China) were added. The absorbance of each well was measured using a microliter enzyme-linked immunosorbent assay reader (iMark^TM^ BIO-RAD) at a wavelength of 490 nm. All experiments were performed in triplicate. The relative survival rate of cells was calculated using the formula: living rate (%) = (A_P_/A_C_) × 100%, where A_C_ and A_P_ are the optical density without (A_C_) and with polysaccharides (A_P_), respectively. When the living rate is over 85%, it is considered that the polysaccharide does not exert toxicity on the living cells [80,81].

### 4.7. Immuno-Modulatory Effect

#### 4.7.1. Mouse Splenic Lymphocyte Proliferation Assay

The spleen was harvested from a Kunming mouse aseptically, and then transferred into a petri dish with PBS. The spleen was minced and blown fully to obtain the cells suspended and filtrated through a 200 meshes steel sieve. A total of 2.5 × 10^4^ cells per well in 96-well plates were treated with three sulfated polysaccharides at a final concentration of 0 μg/mL (control group), 20 μg/mL, 100 μg/mL, and 500 μg/mL followed by incubation for 48 h in a humid atmosphere with 5% CO_2_ at 37 °C. The control group (Mock) used PBS instead of the polysaccharide solution. For each concentration, five repeats were performed. An MTT assay was used to detect the proliferation of lymphocytes. Meanwhile, the lymphocytes proliferation rate was calculated using the formula Proliferation rate (%) = [($\bar{\text{A }}$(test group) − $\bar{\text{A }}$(control group))/ $\bar{\text{A }}$(control group)] × 100% [82]. $\bar{\text{A }}\;$is the average absorbance of the wells with the same treatment.

#### 4.7.2. Animals *Grouping* and *Treatment*

The mice were randomly divided into eight groups with eight mice in each group, including the experimental group, control group, and vaccine group. The experimental groups were given sulfated polysaccharides UPP, GFP and SQP, at doses of 10 mg/kg and 50 mg/kg, mixed with inactivated AIV solution by intraperitoneal injection. Mice in the control group were given the same volume of physiological saline (25 mL/kg) instead. Each mouse in the vaccine group (positive control group) was treated with inactivated AIV H9N2 solution mixed with white oil (100 mg/kg). On Day 1 and 14, the mice were separately immunized (Prime and Boost), and the sera were collected after 14 days of the prime and boost immunizations (on Day 14 and 28), respectively to be saved for the subsequent assays.

#### 4.7.3 AIV-Specific Antibody Titer Detection

Using the sera collected in 4.7.2, the H9N2-specific antibody titers were tested with an ELISA method [83,84], and each treatment was assayed in triplicate. Briefly, a sample or standard was added in ELISA plates coated with capture antibody for 2 h at 37 °C to detect the bound cytokines using a biotinylated anti-cytokine antibody, Avidin HRP, and tetramethylbenzidine. Color development was stopped with 2 M H_2_SO_4_ and optical densities were read at 450 nm.

#### 4.7.4. Cytokines Production

The sera samples collected in 4.7.2 were used to detect cytokines production. Two cytokines (*i.e.*, IL-4 and IFN-γ) in the treated mice were measured with an ELISA kit (Longtun, Shanghai, China) per the manufacturer’s instructions.

#### 4.7.5. T-Cell Subpopulation and Flow Cytometry

The blood cells of the treated Kunming mice were collected, and three-color flow cytometry analyses were performed using different mixtures of specific mAbs, CD3, CD4, and CD8 were labelled with PE-Cy5, FITC or PE, respectively. Data analysis was conducted with the FAC Scan flow cytometer (BD, FACS Aria ii, Franklin Lakes, NJ, USA) using Cell Quest software.

### 4.8. Anti-AIV Effect in Vitro

In the antiviral experiment, the MDCK cells treated with neither AIV nor polysaccharides served as a cell control group, and the cells infected with the virus only served as a virus control group, while the test group was treated with both the virus and polysaccharides. For all groups, MDCK cells were seeded at 2 × 10^5^ cells/well into 24-well plates, and incubated at 37 °C in an atmosphere of 5% CO_2_ until a cell monolayer had formed. The virus control group and test group were treated with 100 μL of the virus solution at a multiplicity of infection (MOI) of 0.2, while for the cell control group, 100 μL of DMEM (1% FBS) was added instead. After incubation for 1 h, the supernatant was discarded, and hereafter, 1 mL of the test polysaccharide solution (GFP, UPP, and SQP, at a concentration of 100 μg/mL and 20 μg/mL, respectively) were added to the test group, while 1 mL of DMEM was added to the virus control and cell control groups. For each group, three biological repetitions were carried out. Treated cells were then incubated at 37 °C with 5% CO_2_ for 24 h.

#### 4.8.1. Virus Titers Assay

Virus titers were determined by the HA test. A total of 25 μL of supernatant from the 24-well plate described above were serially two-fold diluted in saline on a 96-well microtiter V-plate. To each well, 25 μL of 1% red blood cell suspension was added, and the mixtures were gently mixed and incubated at 37 °C for 30 min. HA was observed and judged as positive if more than 50% of the red blood cells were agglutinated in the well [85].

#### 4.8.2. Relative Expression of Viruses

Real-time PCR was applied for quantitative analysis of AIV replication levels *in vivo*. Briefly, total RNA was extracted from the treated MDCK cells in 4.8 section using 1 mL of RNAiso Plus Reagent (Takara BIO INC, Liaoning, China) as per the manufacturer’s instructions. Reverse transcription was immediately performed with Reverse Transcriptase M-MLV (RNase H-; Takara BIO INC, Liaoning, China). Finally, first strand cDNA was used as a template for real-time PCR with a SYBR^®^*Premix ExTaq*^TM^ Kit (Takara BIO INC, Liaoning, China). The primers for AIV were: Sense 5′-ACCAGTGCATGGAGACAATTC-3′ and anti-sense 5′-CAAATGTTGCATCTGCAAGAC-3′; and the primers for internal control β-actin were: Sense 5′-CTGGACTTCGAGCAGGAGATG-3′ and anti-sense 5′-CGGATGTCCACGTCACACTTC-3′. The amplification cycles were carried out as follows: 95 °C for 30 s; 95 °C for 5 s and 60 °C for 34 s (40 cycles). β-actin was used as an internal control to normalize the relative gene expression levels calculated based on the comparative Ct method with the formula 2^−∆∆Ct^ [86].

### 4.9. Statistical Analysis

Data in the figures are expressed as the Mean + SD. A *t-*test was used to analyses the difference among groups with SPSS Software. Differences between means with *p* < 0.05 were considered statistically significant.

## 5. Conclusions

For understanding and comparing the molecular structures and biological activities of algae sulfated polysaccharides, three marine algae *G. filicina*, *U. pertusa* and *S. qingdaoense* were selected from Rhodophyta, Chlorophyta and Ochrophyta, respectively. We characterized the structure determination, the humoral immune responses, and cell immune responses to antiviral assays in this study. According to the results, these three types of sulfate polysaccharides shared chemical characteristics and basic structures, with some variations. Further experiments revealed that the sulfate polysaccharides not only enhanced the immunity level stimulated by inactivated AIV *in vivo* but also significantly inhibited the activity of activated AIV *in vitro*. The results indicated that these sulfated polysaccharides from algae could be a potential immune-stimulant and adjuvant against AIV. Moreover, comparative studies of different species polysaccharides were our focus with the goal of obtaining profound and refined insights into this topic. Sulfated polysaccharides from *S. qingdaoense* had the best immune enhancement bioactivity both *in vitro* and *in vivo*. *G. filicina* sulfated polysaccharides showed the best effect in anti-AIV activity. Comprehensive analyses were performed to determine whether the differences in bioactivities could be explained by the differences in structures. These comprehensive analyses enhanced our understanding of algae sulfated polysaccharides and their potential use in future research but also gave guidance for further selection of immunologic stimulant and anti-viral drugs.

## Appendix

**Table A1:** Relative Survival Rate of the Madin-Darby canine kidney (MDCK) Cells.

Conc (mg/mL)	10	5	2.5	1.25	0.625	0.3125	0.156	0.078
UPP	0.82	1.03	1.16	1.09	1.05	1.08	1.06	1.02
GFP	0.74	0.81	0.89	0.9	0.95	0.92	1.01	1.13
SQP	0.78	0.96	1.01	1	0.97	1.05	0.99	1.03

Determination of the safe concentration by pre-experiment cytotoxicity tests. Cytotoxic activity of the sulfate polysaccharides. The relative living cell rate measured with an MTT assay reflected the cytotoxic activity of the sulfate polysaccharides.
